# Pro-Health and Anti-Cancer Activity of Fungal Fractions Isolated from Milk-Supplemented Cultures of *Lentinus* (*Pleurotus*) *Sajor-caju*

**DOI:** 10.3390/biom11081089

**Published:** 2021-07-23

**Authors:** Adrian Zając, Mateusz Pięt, Dawid Stefaniuk, Michał Chojnacki, Joanna Jakubowicz-Gil, Roman Paduch, Anna Matuszewska, Magdalena Jaszek

**Affiliations:** 1 Department of Functional Anatomy and Cytobiology, Institute of Biological Sciences, Maria Curie-Skłodowska University, 20-031 Lublin, Poland; jjgil@poczta.umcs.lublin.pl; 2 Department of Virology and Immunology, Institute of Biological Sciences, Maria Curie-Skłodowska University, 20-031 Lublin, Poland; piet.mateusz@poczta.umcs.lublin.pl (M.P.); rpaduch@poczta.umcs.lublin.pl (R.P.); 3 Department of Biochemistry and Biotechnology, Institute of Biological Sciences, Maria Curie-Skłodowska University, 20-031 Lublin, Poland; anna.matuszewska@poczta.umcs.lublin.pl (A.M.); magdalena.jaszek@poczta.umcs.lublin.pl (M.J.); 4 Department of Experimental Hematooncology, Medical University of Lublin, 20-031 Lublin, Poland; mkpcho@gmail.com; 5 Department of General Ophthalmology, Medical University of Lublin, 20-031 Lublin, Poland

**Keywords:** fungi, arboreal medicinal mushrooms, *Pleurotus sajor-caju*, *Lentinus sajor-caju*, bioactive compounds, colorectal cancer, anti-cancer activity, milk supplementation

## Abstract

The present study aimed to demonstrate *Lentinus* (formerly *Pleurotus*) *sajor-caju* (PSC) as a good source of pro-health substances. It has also shown that supplementation of its culture medium with cow milk may further improve its beneficial properties. Intracellular fractions from fungi grown on a medium supplemented with cow milk were analyzed using various biochemical methods for determination of the nutrient composition. Furthermore, anti-cancer properties of selected extracts were investigated on colorectal cancer cell lines (HT-29, LS 180, and SW948) in vitro. Biochemical analysis showed enrichment in health-enhancing compounds, such as proteins or polysaccharides (about 3.5- and 4.5-fold increase in concentration of proteins and carbohydratesin extracts of mycelia cultured on whole milk (PSC2-I), respectively), with a decrease in the level of free radicals (10-fold decrease in extract grown on milk and medium mixture (1:1) (PSC3-II)), which was related to increased catalase and superoxide dismutase activity (7.5-fold increase in catalase activity and 5-fold in SOD activity in PSC3-II compared to the control). Moreover, the viability of the cancer cells was diminished (to 60.0 ± 6.8% and 40.0 ± 8.6% of the control, on HT-29 and SW948 cells, respectively), along with pro-apoptotic (to 18.8 ± 11.8 and 14.7 ± 8.0% towards LS 180 and SW948 cells, respectively) and NO-secreting effects (about 2-fold increase) of the extracts. This study suggests that PSC has multiple nutritional and anti-cancer properties and can be used as a source of healthy biomolecules in modern medicine or functional foods.

## 1. Introduction

Fungal material has been used by humankind for centuries, especially due to its gastronomic and pro-health properties. Edible mushrooms are also widely used in daily diet, mainly due to their unique organoleptic properties (characteristic taste and smell), and in ethnomedicine. A significant influence of fungi and their metabolites is particularly evident in natural medicine, especially in Asia. Mushrooms are considered as a very rich source of bioactive compounds. Furthermore, they are easy and cheap to culture and harvest. They can grow in competitive conditions, e.g., in low light, and have many defense mechanisms against pathogens (e.g., production of antibiotics). They produce nutrients and/or medicinal substances in a relatively large amount per dry weight of the fruiting body. Mushrooms are used in contemporary medicine, pharmacy, and dietary supplements. They are also used as functional food. There are various proteins and peptides with health-promoting properties isolated from fungal material, for example, immunomodulatory, antimicrobial, and anti-cancer proteins, as well as lectins, laccases, and ribosome inactivating proteins (RIPs) [1,2,3,4,5,6]. A number of studies demonstrate that many edible mushrooms produce a large proportion of essential amino acids. Therefore, they can successfully substitute meat or animal products in a vegetarian or vegan diet. Furthermore, they produce essential amino acids (phenylalanine, leucine, lysine, tryptophan, methionine, threonine, isoleucine, and valine) in significant amounts and other amino acids, such as arginine or histidine, which may have a beneficial effect on human health [7]. A substantial part of the health-promoting properties of edible mushrooms is attributed to fungal polysaccharides exhibiting antitumor, immunostimulating, immunomodulating, antibacterial, or antiviral properties [6,8]. Triterpenoids are a crucial group of bioactive substances produced by fungi. They were found to have antiviral activity against HIV1 and the herpes virus. Their ability to inhibit cholesterol synthesis has also been confirmed. They lower blood pressure and reduce platelet aggregation, which substantially reduces the risk of cardiovascular diseases [6]. Other compounds with biological activity are phenolics, which are the most abundant components in mushroom fruiting bodies and determine their antioxidant properties. Phenolic compounds of fungal origin possess anti-inflammatory and anti-cancer properties, as well [9,10]. Moreover, mushrooms are investigated not only for their various nutritional properties or bioactive substances contained therein but also for their potential use in the production of functional food [11]. Special attention is paid to the use of the medicinal mushrooms in cow milk processing. Milk exerts health benefit effects due to its abundance of nutrients and bioactive agents, but consumption thereof may cause some allergic reactions and could be harmful for subjects with lactose intolerance. Therefore, mushroom fermentation of cow milk seems to be valuable in further functional food production [12].

The usage of fungi and their prospective anti-cancer properties, especially as functional food, may be of great interest in carcinoma treatment, specifically in colorectal cancer. It is estimated that about half of colorectal cancer cases are caused by lifestyle factors, in particular by improper diet. Therefore, functional food may be potentially beneficial in the prevention of colorectal cancer. This malignancy ranks third in terms of incidence, and the second in mortality. Incidence rates are approximately 4-fold higher in developed countries [13]. The disease usually develops slowly, and symptoms depend, in part, on the location of the cancerous process. It is often diagnosed at a late stage, which significantly reduces the chances of patient survival. Many therapeutic strategies, like the combination of surgery, chemotherapy, radiation, and targeted therapy, often tend to be not efficient enough. Low detection rate, unspecific symptoms, improper diet, and resistance to treatment are what leads to the high mortality rate of the colorectal cancer [14]. Therefore, the usage of mushrooms with potentially pro-health and anti-cancer properties may aid the classical and modern therapies. As mentioned above, colorectal cancer is highly diet-dependent; however, as the colon is the part of the digestive system, colorectal cancer cells are more susceptible to the compounds administered orally.

Considering all of this, our study aimed to investigate the effect of the fungal culture medium supplementation with cow milk on the biochemical composition and anti-cancer activity of *Lentinus sajor-caju* (formerly known as *Pleurotus sajor-caju*) towards colorectal cancer cells.

## 2. Materials and Methods

### 2.1. Strain, Medium, Growth, Processing, and Preparation of Samples

*Lentinus sajor-caju* (formerly *Pleurotus sajor-caju*) deposited under the strain number FCL237 was obtained from the Fungal Collection, Department of Biochemistry and Biotechnology, Maria Curie-Skłodowska University, Lublin, Poland. Fungal cultures were cultivated on Lindeberg-Czapek medium [15]. After four weeks, mycelium was collected and transferred into four medium variant flasks: 1-Lindeberg-Holm medium; 2-commercial whole milk (lactose 5.3%, casein 3.2%, fat 5.5%, total proteins 4.2%); 3-whole milk and Lindeberg-Holm medium 1:1 *v/v*; 4-whole milk and Lindeberg-Holm medium in 1:2 *v/v*. Then, the cultures were harvested after 2 (variant I) or 4 weeks (variant II) of growth. The mycelium was separated from the culture medium and washed with distilled water. Afterwards, 1 g/mL of mycelium was homogenized in a Potter Homogenizer (Sartorius, Goettingen, Germany) (1200 rpm for 5 min), centrifuged, and freeze-dried. The lyophilized samples were weighed and dissolved in sterile RPMI 1640 medium (Sigma Aldrich, St. Louis, MO, USA) to obtain a 100 mg/mL stock solution diluted to the concentrations used in the subsequent analyses.

### 2.2. Cell Cultures

The research was conducted on four cell lines: human normal colon epithelial cells CCD 841 CoTr (ATCC No. CRL-1807), human colon cancer cells HT-29 (ATCC No. HTB-38), human colon cancer cells LS 180 (ATCC No. CCL-187), and human colon cancer cells SW948 (ATCC No. CCL-237). CCD 841 CoTr were cultured in a mixture (1:1 *v/v*) of RPMI 1640 and DMEM media (Sigma) supplemented with 10% FBS Gibco (Thermo Fisher Scientific), Waltham, MA, USA)and antibiotics (100 U/mL penicillin, 100 µg/mL streptomycin) (Gibco) and kept at 34 °C in a humidified atmosphere with 5% CO_2_. HT-29, LS 180, and SW948 cells were cultured in RPMI 1640 medium supplemented with 10% FBS and antibiotics and kept at 37 °C in a humidified atmosphere with 5% CO_2_ flow The cell lines were selected to represent different stages and grades of colorectal cancer: HT-29-stage I, grade II, LS 180-stage II, grade II, SW948-stage III, grade III. Furthermore, these cell lines have a different profile of key genes and proteins related during carcinogenesis: mutations in oncogenes and suppressor genes (*TP53* (HT-29 and SW948), *KRAS* (LS 180 and SW948), *BRAF* (HT-29), *PI3KCA* (HT-29, LS 180, SW948), microsatellite instability (LS 180), chromosomal instability (HT-29, SW948), CpG island methylation phenotype (CIMP) (HT-29) [16,17,18,19]. As a control to our studies, CCD 841 CoTr (human normal colon epithelial) cell line was used.

### 2.3. Evaluation of Biochemical Properties

#### 2.3.1. Determination of Amino Acids, Proteins, Carbohydrates, and Phenolic Compounds

The ninhydrin method was employed to assess the concentration of free amino acids. The amount of free amino acids was evaluated spectrophotometrically with glycine as standard substrate [20]. Protein concentrations were determined using the Bradford reagent and bovine serum albumin as a standard [21,22]. Assessment of total sugar content was performed with the phenol-sulfuric acid assay using D-glucose as a standard [23,24]. The complete range of phenolic compounds was determined with diazosulfanilamide using the DASA test, where absorbance was measured at 500 nm, and vanillic acid was used as a standard [24,25].

#### 2.3.2. Determination of Free Radicals

The level of free radicals was evaluated by the NBT assay according to the Malarczyk and Wilkołazka method. Mycelial samples were mixed with 50 µL of 4% nitro blue tetrazolium, 250 µL 1N NaOH, and 1,5 mL H_2_O and incubated at room temperature for 30 min. Afterwards, the samples were measured spectrophotometrically at 560 nm [26,27].

#### 2.3.3. Electrophoretic Assessment of Catalase and Superoxide Dismutase Activity

Homogenized mycelia were used for electrophoretic analysis. Electrophoretic separation was carried out on 12.5% polyacrylamide gel, with 25 µL of samples loaded per well. The electrophoresis was held at 4 °C, 140 V. Densitometric semi-quantitative analyses of electropherograms were performed with ImageJ software ver. 1.52a (NIH, Bethesda, MD, USA). For analysis of catalase activity, gels were rinsed 3 times in distilled water and incubated in 0.003% H_2_O_2_ for 10 min. The gels were rinsed and placed in a mixture of equal volumes of 2% K_2_Fe(CN)_6_ and 2% FeCl_3_ and incubated for 10 min. Superoxide dismutase activity was measured using the NBT method. The gels were incubated for 30 min with NBT (nitrotetrazolium blue), rinsed, and placed in a mixture of methionine, riboflavin, and K_2_HPO_4_ [28,29].

### 2.4. Assessment of Anti-Cancer Activity

#### 2.4.1. MTT Assay

The MTT method is based on reducing yellow salt (MTT, Sigma Aldrich, St. Louis, MO, USA) to purple formazan crystals, which is catalyzed by enzymes present mainly in the endoplasmic reticulum. The absorbance of diluted formazan crystals is directly proportional to the number of living cells. After 24-h incubation of the cells in a 96-well plate (100 µL of 1 × 10^5^ cells/mL) with the studied extracts, 25 µL of 5 mg/mL MTT (Sigma Aldrich, St. Louis, MO, USA) were added to each well. After 3 h, 100 µL of 10% SDS (Sigma Aldrich, St. Louis, MO, USA in 0.01 M HCl (POCH, Gliwice, Poland) were added and incubated for 24 h. The plates were read at 570 nm using a Microplate Reader (BioTek, Winooski, VT, USA).

#### 2.4.2. NR Uptake Assay

In the NR method, after 24-h incubation of the cells in 96-well plate (100 µL of 1 × 10^5^ cells/mL) with the studied extracts, the medium was removed and 100 µL of 40 mg/mL NR were added to each well. After 3-h incubation, the cells were fixed with 200 µL of 0.5% formalin in 1% CaCl_2_. Subsequently, 100 µL of solvent (1% acetic acid in 50% ethanol) were added for 20 min. The plates were measured at 540 nm.

#### 2.4.3. May-Grünwald—Giemsa Staining

Cells at a density of 1 × 10^5^ cells/mL were cultured in Petri dishes (35 mm). After 24-h incubation with the studied extracts, the medium was discarded, and the MGG staining protocol was initiated. The cells were stained and fixed with the May-Grünwald dye for 3 min and another 3 min in the dye diluted with an equal volume of water. Thereafter, the dye was removed and Giemsa stain, previously diluted (1:19 in water), was added for 20 min. The dishes were rinsed three times with distilled water and dried. The observation was performed under a light microscope Olympus BX51 (Olympus, Tokio, Japan) with 100× magnification and analyzed. Changes in the number and morphology of the cells were verified.

#### 2.4.4. Nitric Oxide Secretion Measurement

The indirect measurement of NO was conducted using the spectrophotometric method based on the Griess method.

Cells at a density of 1 × 10^5^ cells/mL were cultured in 24-well plates. After 24-h incubation with the studied extracts (with or without 2-h pre-incubation with *E. coli* LPS (10 μg/mL) (Sigma Aldrich, St. Louis, MO, USA) the medium was collected and frozen at −80 °C. For the analysis, the samples were thawed, and 100 μL of samples and standards (1, 5, and 10 μM of NaNO_2_ (Sigma Aldrich, St. Louis, MO, USA) were added to the wells of the 96-well plate. Afterwards, 100 μL of Griess reagent (1% sulfanilamide/0.1% N-(1-naphthyl) ethylenediamine dihydrochloride (POCH, Gliwice, Poland) in 3% H_3_PO_4_ (POCH, Gliwice, Poland) were added to each well. After 10-min incubation at room temperature, the plates were read using a Microplate Reader, and absorbance was measured at 570 nm.

#### 2.4.5. Apoptosis Evaluation-Hoechst 33342/PI Staining

Evaluation of apoptosis and necrosis in the cell cultures was performed by staining with DNA-intercalating fluorochromes: Hoechst 33342 (Sigma Aldrich, St. Louis, MO, USA) and propidium iodide (PI) (Sigma Aldrich, St. Louis, MO, USA). Cells exhibiting blue fluorescence of nuclei (fragmented or with condensed chromatin) were interpreted as early apoptotic. Morphologically similar cells with pink fluorescence were defined as late apoptotic. Enlarged cells with pink fluorescence of whole nuclei were classified as necrotic. 

Cells at a density of 1 × 10^5^ cells/mL were cultured in Petri dishes (35 mm). After 24-h incubation with the extracts at a concentration of 200 μg/mL, 5 μL of a Hoechst 33342 and PI mixture were added to the medium in each dish. The samples were incubated for 5 min, washed with PBS, and observed under a fluorescence microscope (Olympus BX51, Tokio, Japan ) at magnification 100×. More than 1000 cells in randomly selected areas were counted, and the ratios of apoptotic and necrotic cells were assessed.

#### 2.4.6. DAPI/SR101 Staining

To visualize morphological changes in the nuclei and protein localization, DAPI/SR101 staining was performed. The blue-fluorescent DAPI stained cell nuclei, and the red-fluorescent SR101stained cell proteins.

After-24 h incubation with the studied extracts (200 μg/mL), the cells were fixed and incubated with a DAPI/SR101 (Sigma Aldrich, St. Louis, MO, USA) mixture. The cells were observed and photographed at magnifications 100× (for normal cells) and 200× (for cancer cells) at 460–500 nm (DAPI) and 600 nm (SR101) using a fluorescence microscope Olympus BX51. The intensity of the red fluorescence of proteins was evaluated with ImageJ software ver. 1.52a (NIH, Bethesda, MD, USA). Six random areas were measured from every image; results from 3 repeats were presented as mean ± SD.

### 2.5. Statistical Analysis

All analyses were performed in at least 3 replications, and the data were analyzed using GraphPad Prism ver. 7.01 (GraphPad Software, San Diego, CA, US). The results were presented as mean ± SD. Statistical significance was evaluated with the one-way Anova test and post-hoc Dunnett’s test.

## 3. Results

### 3.1. Screening Selection of Samples

During the screening analysis, three extracts of *L. sajor-caju* were selected for further analysis: PSC2-I, PSC3-II, and PSC4-II. These were extracts cultured on whole milk only for 2 weeks (PSC2-I), cultured on whole milk and Lindeberg-Holm medium 1:1 *v/v* for 4 weeks (PSC3-II), and cultured on whole milk and Lindeberg-Holm medium 1:2 *v/v* for 4 weeks (PSC4-II). The screening was performed on CCD 841 CoTr and HT-29 cells using the NR method. The extracts were selected due to their stronger effect towards cancer cells with a concurrently milder effect on normal cells. The biochemical properties of extracts from the fungus grown on milk or milk-supplemented medium were compared to the control, i.e., fungal mycelium cultured on the standard mineral medium (Lindeberg-Holm) for 2 weeks (control for PSC2-I) and 4 weeks (control for PSC3-II and PSC4-II), respectively. The controls are presented as green bars in the figures in the biochemical section. The control in the cell culture section for the anticancer activity investigations were cells cultured in the medium with FBS only (with no extracts added).

### 3.2. Biochemical Composition of Extracts

#### 3.2.1. Determination of Proteins, Amino Acids, Carbohydrates, Phenolic Compounds, and Free Radicals

**Figure 1:**
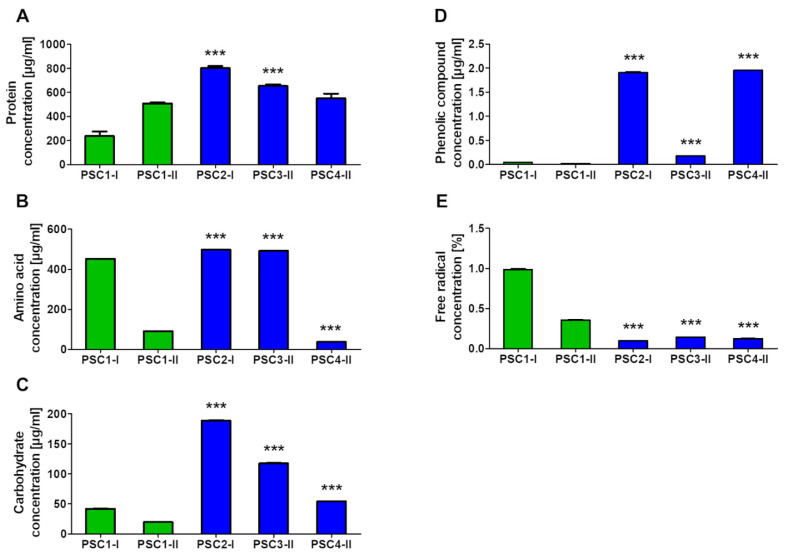
Biochemical composition of the PSC2-I, PSC3-II, and PSC4-II extracts compared to the control (PSC1-I for PSC2-I, and PSC1-II for PSC3-II and PSC4-II): concentration of proteins (**A**), amino acids (**B**), carbohydrates (**C**), phenolic compounds (**D**), level of free radicals (**E**); *** *p* < 0,005, one-way ANOVA, Dunnett’s test.

The determination of proteins showed that all selected extracts had a higher protein concentration than the control (Figure 1A). The level of free amino acids (aa) determined in the extracts was lower than in the control, with the lowest aa concentration in PSC4-II (Figure 1B). The analysis of carbohydrates showed that the PSC2-I and PSC3-II extracts had a significantly higher level of these substances than the control. In turn, the concentration of carbohydrates in the PSC4-II extract was lower than in the control (Figure 1C). The analysis of the level of phenolic compounds revealed their significantly higher concentrations in all selected extracts, compared to the control (Figure 1D).

The analysis of the concentration of free radicals revealed their significantly lower levels in all extracts in comparison with the control. The lowest level was determined in PSC2-I, compared to the other extracts. In turn, the highest free radical level was determined in PSC3-II; however, their concentration was lower than in the control (Figure 1E). The concentrations of the compounds are presented in the (Supplementary Materials (Table S1)).

#### 3.2.2. Electrophoretic Analysis of Catalase and SOD Activity

**Figure 2:**
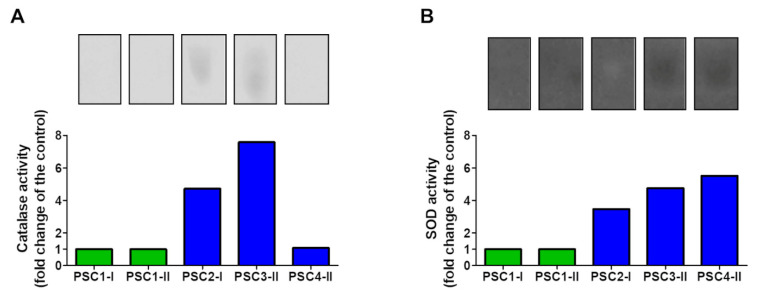
Biochemical properties of the PSC2-I, PSC3-II, and PSC4-II extracts compared to the control (PSC1-I for PSC2-I, and PSC1-II for PSC3-II and PSC4-II): activity of catalase (**A**) and SOD (**B**).

The electrophoretic analysis of catalase activity revealed that all studied extracts increased the enzyme activity several-fold, compared to the controls. The strongest effect was exhibited by PSC3-II (Figure 2A).

Similarly, SOD activity was also several-fold increased by all the extracts, with the highest effect exerted by PSC4-II (Figure 2B).

### 3.3. Evaluation of Anti-Cancer Properties

#### 3.3.1. Cytotoxicity Assessment

**Figure 3:**
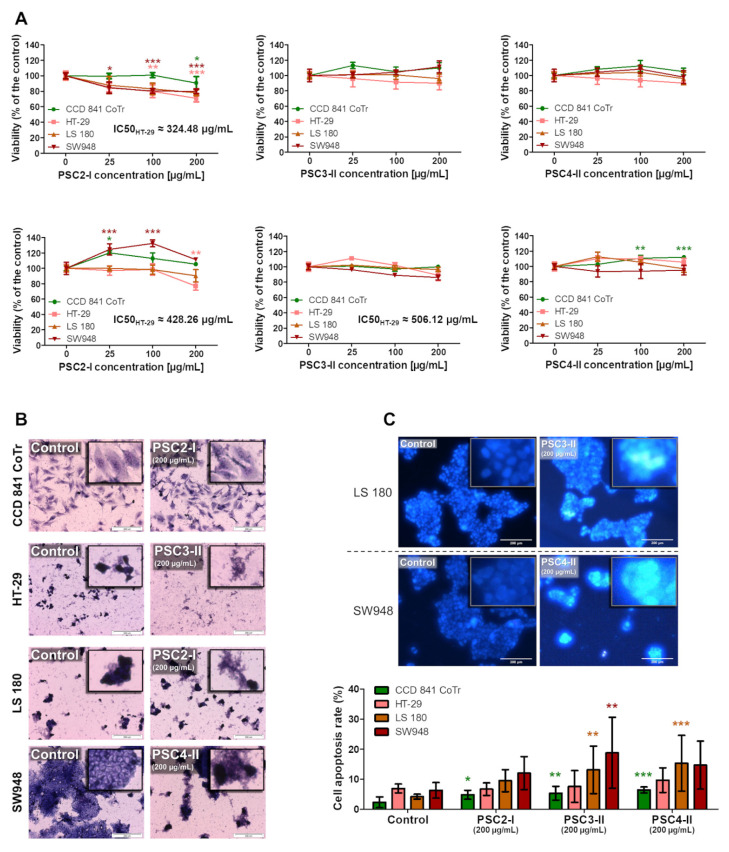
Anti-cancer effect of the PSC2-I, PSC3-II, and PSC4-II extracts on CCD 841 CoTr, HT-29, LS 180, and SW948 cells: (**A**) effect on cell viability (upper panel: NR method, PSC2-I, PSC3-II, and PSC4-II in the row, respectively; bottom panel: MTT method, PSC2-I, PSC3-II, and PSC4-II in the row, respectively); (**B**) effect of the extracts (at 200 µg/mL) on cell morphology-May-Grünwald-Giemsa staining (for all images, see Supplementary Materials Figure S1) (the measurement bars indicate 200 µm); (**C**) effect of the extracts (at 200 µg/mL) on the apoptotic rate (the measurement bars indicate 200 µm) (for all images, see Supplementary Materials Figure S2); * *p* < 0,05, ** *p* < 0,01, *** *p* < 0,005, one-way ANOVA, Dunnett’s test.

The NR method indicated that only PSC2-I showed significant cytotoxic activity. It inhibited the viability of HT-29 in a dose-dependent manner. The IC_50_ value was approximately 323.3 μg/mL. The extract also inhibited proliferation of CCD 841 CoTr and SW948 cells. LS 180, however, seemed to be invulnerable (Figure 3A, upper panel). The results obtained with the MTT method appear slightly different. They showed that the effect of the extracts was associated with disruption of cell metabolism rather than disruption of membrane integrity. The extract reduced the viability of all examined cell lines, excluding HT-29. However, the viability reduction in the CCD 841 CoTr cells was no higher than 10% (Figure 3A, bottom panel). The images produced by May-Grünwald-Giemsa staining confirm the NR and MTT assays; there were visible morphological changes in the cells, as well as dead cells, compared to the control (Figure 3B). The PSC3-II and PSC4-II extracts did not exert a significant cytotoxic effect. They induced proliferation or inhibited viability to a small extent (excluding the effect of PSC4-II on CCD 841 CoTr measured with the MTT method (Figure 3A, upper panel). The results were confirmed by staining the cells with the May-Grünwald-Giemsa method. There were slightly greater numbers of morphologically changed and dead cells compared to the control, but the changes were not distinct (Figure 3B).

#### 3.3.2. Apoptosis and Necrosis Evaluation

All studied extracts exhibited the desired activity. They increased the apoptosis rate in all cancer lines with no significant rise in apoptotic CCD 841 CoTr cells. Furthermore, the higher the stage of cancer was, the higher the pro-apoptotic effect was. The most pronounced effect was exerted by PSC4-II on HT-29 and LS 180 and by PSC3-II on SW948 (Figure 3C). The cell necrosis rate was insignificant, i.e., lower than 1% in most cases (with the highest necrotic rate of 1.1% in the SW948 cells incubated with PSC2-I).

#### 3.3.3. Evaluation of Morphological Changes in Nuclei and the Concentration and Localization of Proteins

No significant changes in the CCD 841 CoTr cells after the incubation with the extracts were observed.

**Figure 4:**
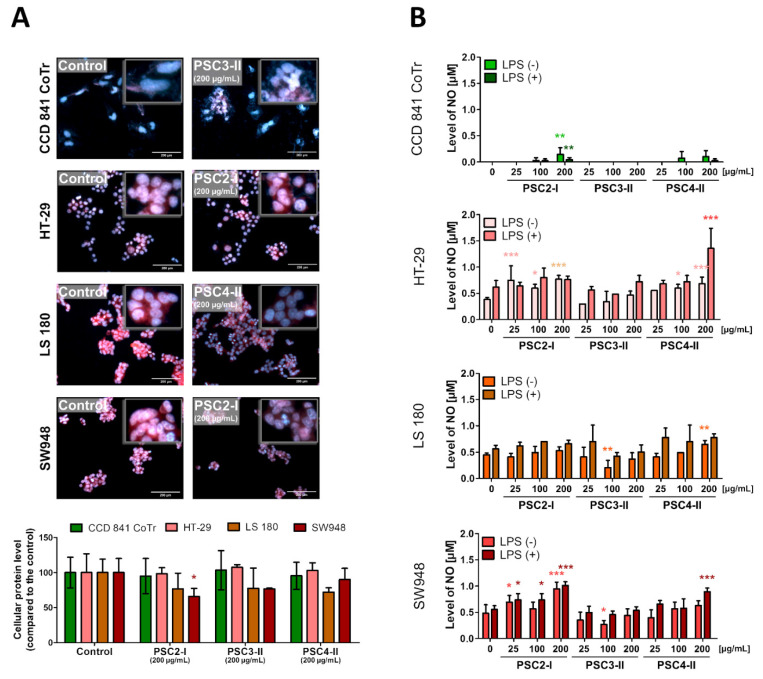
Anti-cancer effect of the PSC2-I, PSC3-II, and PSC4-II extracts on CCD 841 CoTr, HT-29, LS 180, and SW948 cells: (**A**) effect on the morphology of the cells and level of proteins measured with DAPI/SR101 staining (upper panel; the measurement bars indicate 200 µm; for all images, see Supplementary Materials Figure S3) and level of the cellular proteins determined through measurement of the red fluorescence intensity (bottom panel) after incubation with extracts at 200 µg/mL; (**B**) concentration of NO secreted by the cells exposed to the studied extracts measured with the Griess method (exact concentrations are presented in Supplementary Materials Tables S2–S4); * *p* < 0,05, ** *p* < 0,01, *** *p* < 0,005, one-way ANOVA, Dunnett’s test.

The effect of the PSC2-I, PSC3-II, and PSC4-II extracts on cancer cells included morphological changes, e.g., enlarged cells probably with cytoplasm leakage (potentially cells in the state of necrosis) and deformed or enlarged nuclei. There were also fewer cells undergoing division. The red color was less intense, which may indicate cytoplasm leakage (with proteins), probably caused by disruption of cell membrane integrity or a decrease in the protein concentration. The strongest anti-cancer effect was exhibited by PSC2-I and PSC3-II on the SW948 cells and by all extracts on the LS 180 cells. The HT-29 cells, however, seemed unaffected (Figure 4A).

#### 3.3.4. NO Secretion Measurement

The cytotoxic effect of the studied extracts was reflected in a rise in the level of the secreted NO. It has been demonstrated that agents inducing NO leakage have the highest cytotoxicity [30,31]. The results are presented in Figure 4B and in Supplementary Materials Tables S2–S4.

The effect on the CCD 841 CoTr cells was mild; the strongest activity was exhibited by PSC2-I at 200 µg/mL in LPS(-) and LPS(+) conditions. The effect on the cancer cells was even more evident. PSC2-I and PSC4-II caused NO secretion by the HT-29 and LS 180 cells in both LPS-presence and LPS-absence conditions. The strongest effect in the absence of LPS was exhibited by PSC2-I at 200 µg/mL in the case of the HT-29 and LS 180 cells. In the case of LPS pre-treatment, the strongest activity was exhibited by 200 µg/mL PSC4-II towards the HT-29 cells and PSC2-I and PSC4-II (both at 200 µg/mL) towards the LS 180 cells. The effect on the SW 948 cells was milder, and the only statistically significant effect was exerted by 200 µg/mL PSC4-II in the absence of LPS.

On the other hand, a drastic decrease in the NO level may also be regarded as anti-cancer activity. Such activity was exhibited by 100 µg/mL PSC3-II towards LS 180 and SW948.

## 4. Discussion

A significant increase in lifestyle diseases has been observed over the past decades. Modern medicine is not always efficient enough and alternatives are being sought. Therefore, the interest in agents used in folk medicine for centuries is growing today. Many of such substances, especially those derived from mushrooms, exhibit pro-health and anti-cancer properties [32,33,34].

The present study demonstrated the biochemical composition and anti-cancer effects of extracts from the arboreal medicinal fungus *Lentinus sajor-caju* cultivated on milk-supplemented medium on human colon cancer cells HT-29, LS 180, and SW948, and on human normal epithelial cells CCD 841 CoTr as a control.

So far, this species has not been studied widely in this regard or has not been studied at all. In turn, the effect of supplementation of culture media with cow milk on anti-cancer activity has been evaluated. Cow milk is an extremely widespread product in the diet of modern society. However, milk consumption raises considerable controversy and seems to be a source of health problems related to allergic reactions to its ingredients in a certain group of consumers. The research presented in our work shows new possibilities of using its potential. Previous research conducted by our team for the first time showed the role of fungal extracts of *Cerrena unicolor* and *Pycnoporus sanguineus* as health-related factors used to improve the pro-health properties of cow milk and its effect on extract activity [35].

The fungus was grown on normal medium (1), medium supplemented with milk (2 and 3), and milk only (4) for two (I) or four weeks (II). After this time, the extracts from all variants were prepared and biochemical analysis was performed, followed by evaluation of the activity of the extracts towards cancer and normal cells. PSC2-I exhibited the best anti-cancer properties, inducing a cytotoxic effect towards cancer cells, increasing the apoptosis ratio, and causing morphological and physiological changes in the cells.

The PSC2-I extract had different biochemical properties than the other extracts and the control. It showed the highest concentration of proteins, free amino acids, carbohydrates, and phenolic compounds and the lowest level of free radicals compared to the other extracts. It also exhibited the strongest anti-cancer properties. The present results may indicate a correlation between these properties. Probably, PSC2-I produced antitumor proteins, thus the cytotoxic effect on the cancer cell lines was observed. On the other hand, the extract promoted proliferation of the normal cells, which was demonstrated by the NR and MTT methods.

Fungal proteins are known for their anti-cancer properties. Lin et al. (2010) studied antitumor and immunomodulatory effects of protein isolated from *Ganoderma microsporum* towards lung cancer A549 cells. It appeared that the protein, called GMI (*Ganoderma microsporum* immunomodulatory), inhibited epidermal growth factor-mediated invasion of cancer cells [36]. PSC2-I and PSC4-II also exhibited elevated levels of phenolic compounds, i.e., substances with confirmed anti-cancer properties, in contrast to PSC3-II. Many phenolic and polyphenolic compounds, such as flavonoids, tannins, lignans, and stilbens, occur in fungal cells. Due to their structure and chemical properties, phenolic compounds have high antioxidant potential [37,38,39]. The antioxidant properties of phenols constitute one of the mechanisms that protect cells against neoplastic changes. They may also exhibit cytotoxic properties, e.g., hericenons A and B from *Hericium erinaceus* on HeLa cells [40]. Gallic acid, i.e., an organic phenolic acid derived from fungi, has been demonstrated to exhibit antitumor properties against prostate cancer and human glioblastoma cells [41,42,43]. It was also found that cinnamic acid isolated from *Clitocybe alexandri* inhibited cell growth in non-small cell lung cancer (NCI-H460), colon cancer (HCT15), colon adenocarcinoma (CaCo-2), and cervical cancer (HeLa) [44,45,46]. Furthermore, polyphenolic compounds are linked to scavenging reactive oxygen forms. We demonstrated that the extracts exhibited potential to reduce the level of free radicals, especially PSC2-I and PSC4-II. Such an effect may be related to the higher level of phenolic compounds and the higher CAT and SOD activity. Similar effects of *Lentinula edodes* and *Lentinus sajor-caju* on HEp-2 and HeLa (cervical cancer) and *L. sajor-caju* on HeLa and HepG2 (hepatocellular cancer) were demonstrated by Finimundy et al. [47,48]. In another study conducted by Seedevi et al., free radical scavenging activity was exhibited by polysaccharide isolated from *L. sajor-caju* [49].

In the present study, we demonstrated an anti-cancer effect of PSC extracts on colon cancer cells HT-29, LS 180, and SW948. A decrease in viability, induction of apoptosis, and morphological changes were observed. Cytotoxic and pro-apoptotic effects of *L. sajor-caju* extracts were demonstrated by Finimundy et al.: the extract decreased the viability of HCT-116 cells, induced apoptosis, and affected the cell cycle, leading to its arrest in the G2/M phase [50]. Chaturvedi et al. reported similar results revealing cytotoxic and pro-apoptotic effects of PSC water extract on HCT-116 cells [51]. The fungus was studied on other models, as well, and was shown to affect human cervical cancer HEp-2 and HeLa cells [47], breast cancer MCF-7 and non-small cell lung cancer NCI-H460 cells [48], and human gastric cancer (AGS) and hepatocellular carcinoma cells (HepG2) [49]. Many more mushrooms and derived compounds have been demonstrated to possess anti-cancer activity. For instance, sesquiterpene Hirsutanol A from *Chandrostereum sp*. had an effect on SW620 cells [52], diterpene cyathin Q from *Cyathus africanus* on HCT116 cells [53], *Lactarius flavidulus* and *Ganoderma capense* on hepatoma HepG2 and leukemic L1210 cells [54,55], and *Pholiota adiposa* and *Hericum erinaceum* on hepatoma HepG2 and breast cancer MCF7 cells [56,57].

Another activity responsible for the PSC anti-cancer effect may be related to an increased NO concentration. Nitric oxide may act as a pro-carcinogenic agent inducing mutagenesis, proliferation, angiogenesis, and metastasis; on the other hand, the anti-cancer effect of NO achieved mainly through apoptosis induction is known, as well. It is thought that low concentrations of nitric oxide are pro-cancerous, while higher concentrations favor the anti-cancer effect [30,31]. Therefore, a number of compounds capable of NO releasing or inducing its production are being developed as anti-cancer agents [31,58]. We demonstrated that extracts PSC2-II and PSC4-II had the strongest activities associated with a decrease in cell viability and an increase in NO secretion. Similar results were obtained by Chaturvedi et al. (2020), who demonstrated a rise in the level of intracellular ROS after treatment of HCT-116 cells with PSC extract [51].

A very important aspect of the present study was to evaluate the effect of various culture conditions: time of growth and composition of the medium-the presence (and amount) or absence of cow milk on biochemical and anti-cancer properties. We have previously demonstrated such a correlation in the case of *Cerrena unicolor* and *Pycnoporus sanguineus* [35]. A similar correlation was observed in the case of *Lentinus* *sajor-caju*. The shorter the incubation with milk was (variant I, 2 weeks of culturing on milk), the milder the effect on both normal and cancer cells was. In turn, the longer the growth was (variant II, 4 weeks of culturing of milk), the stronger the effect on both models was exerted. It appears that the best properties are acquired by the mushroom during shorter culturing on milk only (2 weeks, variant I) or longer growth on milk mixed with medium (4 weeks, variants II). Probably, prolonged fermentation of milk by PSC contributes to accumulation of cytotoxic agents with limited specificity (cytotoxic effect towards both normal and cancer cells). Alternatively, at a specific time point, the fungus assimilates pro-health agents, which are fermented or transformed beyond that point. This question is to be answered in future studies.

In the present study, we demonstrated the biochemical composition and anti-cancer properties of *Lentinus* *sajor-caju* extracts, and the effect of culturing the fungus on full cow milk. The results seem to suggest that the mushroom has anti-cancer activity, which is enhanced by cultivation on milk. However, the exact mechanisms responsible for such activity are to be explained in the future.

## 5. Conclusions

In summary, we presented the potential beneficial effects of supplementation of *Lentinus* *sajor-caju* growth medium with milk. We have demonstrated the reducing effect of mycelium extracts on the survival of selected tumor cell lines (HT-29, LS 180, and SW948). All tested extracts exerted an anti-cancer influence through reduction of cancer cell viability, a pro-apoptotic effect, disruption of cell membrane integrity and nuclei morphology, and an increase in NO secretion. There is no doubt that the above results require further and more comprehensive analyses; however, they have revealed the most desired pro-health and anti-cancer properties of the extracts. We have also shown a novel application of the nutritional potential of milk fermented by mushrooms.

## Data Availability

The data presented in this study are available on request from the corresponding author. The data are not publicly available due to privacy.

## Supplementary Materials

The following are available online at https://www.mdpi.com/article/10.3390/biom11081089/s1, Figure S1. Results of screening PSC extracts on cells—cytotoxicity of PSC1-I (A), PSC2-I (B), PSC3-I (C), PSC4-I (D), PSC1-II (E), PSC2-II (F), PSC3-II (G), PSC4-II (H) on HT-29 cells measured with NR method. For the further analyses, PSC2-I, PSC3-II and PSC4-II were selected due to the strongest effect on HT-29 and the weakest cytotoxicity towards CCD 841 CoTr cell (not shown), Figure S2. Anti-cancer effect of the PSC2-I, PSC3-II and PSC4-II extracts on CCD 841 CoTr, HT-29, LS 180 and SW948 cells: effect of the extracts on cell morphology—May-Grünwald- Giemsa staining, Figure S3. Anti-cancer effect of the PSC2-I, PSC3-II and PSC4-II extracts on CCD 841 CoTr, HT-29, LS 180 and SW948 cells: pro-apoptotic effect of the extracts, Figure S4. Anti-cancer effect of the PSC2-I, PSC3-II and PSC4-II extracts on CCD 841 CoTr, HT-29, LS 180 and SW948 cells: effect on the morphology of the cells and level of the proteins measured with DAPI/SR101 staining, Table S1. Biochemical composition of the cow whole milk and the studied extracts of *Pleurotus sajor-caju*, Table S2. Concentration of the NO secreted by CCD 841 CoTr, HT-29, LS 180 and SW948 cells after incubation with PSC2-I extract, with and without pre-incubation with LPS, Table S3. Concentration of the NO secreted by CCD 841 CoTr, HT-29, LS 180 and SW948 cells after incubation with PSC3-II extract, with and without pre-incubation with LPS, Table S4. Concentration of the NO secreted by CCD 841 CoTr, HT-29, LS 180 and SW948 cells after incubation with PSC4-II extract, with and without pre-incubation with LPS.

## Abbreviations

aa	amino acids
LPS	lipopolysaccharides
MGG	May-Grünwald-Giemsa
MTT	(3-(4,5-dimethyl-2-thiazolyl)-2,5-diphenyl-2H-tetrazolium bromide)
NBT	nitrotetrazolium blue
NO	nitric oxide
NR	neutral red dye
PBS	phosphate-buffered saline
PI	propidium iodide
PSC	Lentinus sajor-caju (Pleurotus sajor-caju)
PSC2	I-extract cultured on whole milk only for 2 weeks
PSC3	II-extract cultured on whole milk and Lindeberg-Holm medium 1:1 *v/v* for 4 weeks
PSC4	II-extract cultured on whole milk and Lindeberg-Holm medium 1:2 *v/v* for 4 weeks
RIPs	ribosome inactivating proteins
SOD	superoxide dismutase
SR101	sulforhodamine 101
