# Supplementary material for: Pro-Health and Anti-Cancer Activity of Fungal Fractions Isolated from Milk-Supplemented Cultures of Lentinus (Pleurotus) Sajor-caju
Source: Biomolecules. 2021 Jul 23;11(8):1089. doi: 10.3390/biom11081089 (PMC8391637; doi:10.3390/biom11081089)
Supplement: Supplementary file 1 [file biomolecules-11-01089-s001.zip › biomolecules-1290755-supplementary.pdf]

biomolecules

MDPI

Supplementary material

Prohealth and anticancer activity of fungal fractions isolated from milk-supplemented cultures of *Pleurotus sajor-caju*

Adrian Zając [1,*], Mateusz Pięt [2], Dawid Stefaniuk [3,*], Michał Chojnacki [4], Joanna Jakubowicz-Gil [1], Roman Paduch [2] and Magdalena Jaszek [3]

1 Department of Functional Anatomy and Cytobiology, Institute of Biological Sciences, Maria Curie-Skłodowska University, Lublin, Poland
2 Department of Virology and Immunology, Institute of Biological Sciences, Maria Curie-Skłodowska University, Lublin, Poland
3 Department of Biochemistry and Biotechnology, Institute of Biological Sciences, Maria Curie-Skłodowska University, Lublin, Poland
4 Department of Experimental Hematooncology, Medical University of Lublin, Poland
* Correspondence: adrian.zajac@poczta.umcs.lublin.pl; dawid.stefaniuk@poczta.umcs.lublin.pl

Table S1. Biochemical composition of the cow whole milk and the studied extracts of *Pleurotus sajor-caju*.

**Proteins [µg/mL]**					
**Milk**	**PSC1-I**	**PSC1-II**	**PSC2-I**	**PSC3-II**	**PSC4-II**
523.3 ± 5.3	238.3 ± 36.7	508.3 ± 9.1	802.9 ± 16.6	654.2 ± 12.4	550.5 ± 38.4
**Amino acids [µg/mL]**					
**Milk**	**PSC1-I**	**PSC1-II**	**PSC2-I**	**PSC3-II**	**PSC4-II**
509.5 ± 0.7	452.4 ± 0.1	90.6 ± 0.4	498.9 ± 0.2	492.4 ± 0.5	37.5 ± 0.3
**Carbohydrates [µg/mL]**					
**Milk**	**PSC1-I**	**PSC1-II**	**PSC2-I**	**PSC3-II**	**PSC4-II**
69.5 ± 0.1	42.0 ± 0.2	19.7 ± 0.4	188.6 ± 0.8	117.9 ± 0.5	54.4 ± 0.2
**Phenolic compounds [µg/mL]**					
**Milk**	**PSC1-I**	**PSC1-II**	**PSC2-I**	**PSC3-II**	**PSC4-II**
0.0016 ± 0.0001	0.0447 ± 0.0004	0.0175 ± 0.0003	1.9068 ± 0.0121	0.1749 ± 0.0043	1.9532 ± 0.0029
**Free radicals [%]**					
**Milk**	**PSC1-I**	**PSC1-II**	**PSC2-I**	**PSC3-II**	**PSC4-II**
0.446 ± 0.003	0.987 ± 0.007	0.357 ± 0.002	0.098 ± 0.001	0.144 ± 0.000	0.124 ± 0.001

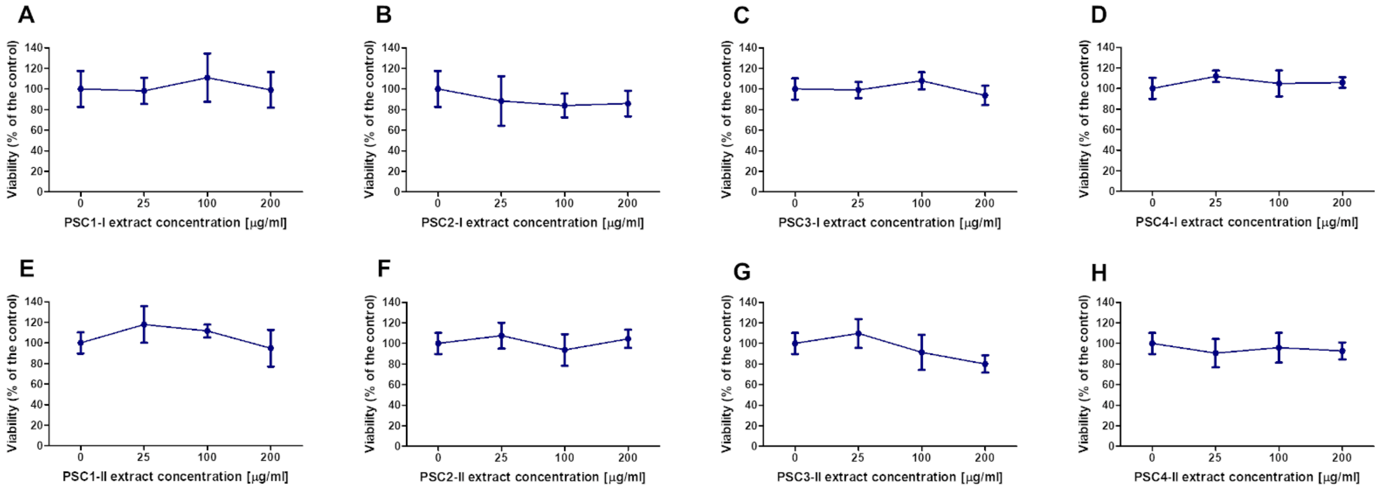

Figure S1. Results of screening PSC extracts on cells – cytotoxicity of PSC1-I (A), PSC2-I (B), PSC3-I (C), PSC4-I (D), PSC1-II (E), PSC2-II (F), PSC3-II (G), PSC4-II (H) on HT-29 cells measured with NR method. For the further analyses, PSC2-I, PSC3-II and PSC4-II were selected due to the strongest effect on HT-29 and the weakest cytotoxicity towards CCD 841 CoTr cell (not shown).

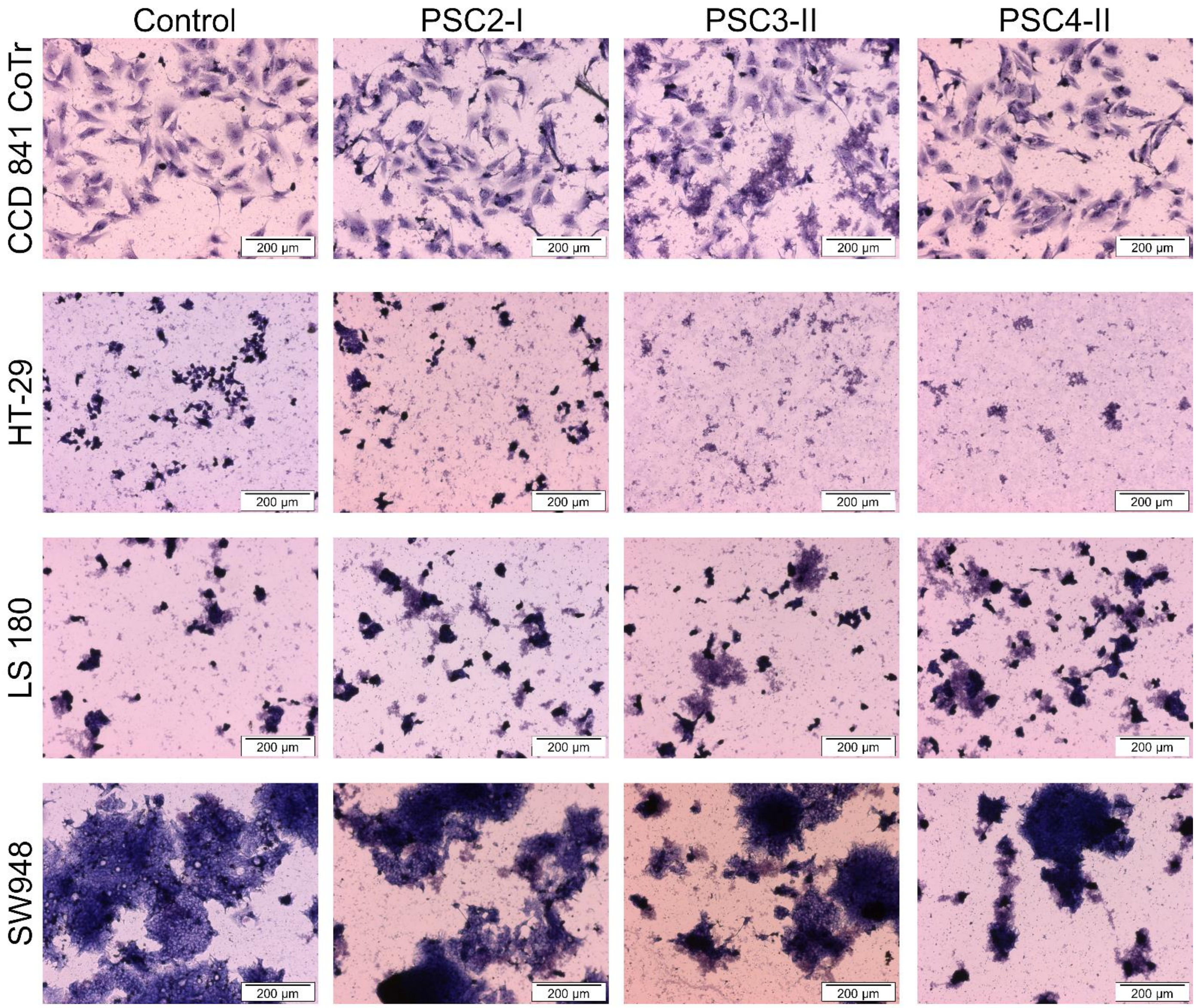

Figure S2. Anti-cancer effect of the PSC2-I, PSC3-II and PSC4-II extracts on CCD 841 CoTr, HT-29, LS 180 and SW948 cells: effect of the extracts on cell morphology – May-Grünwald- Giemsa staining.

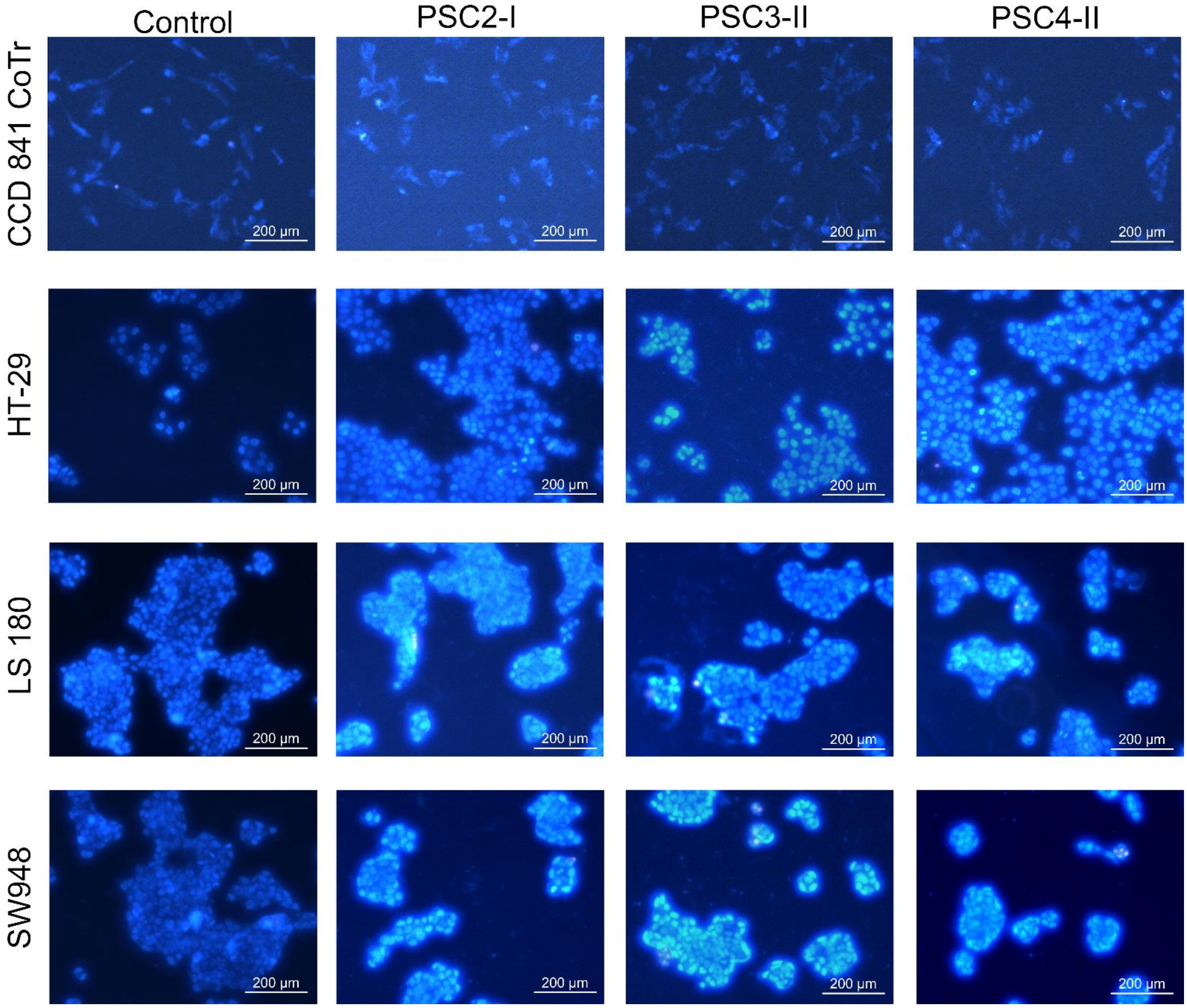

Figure S3. Anti-cancer effect of the PSC2-I, PSC3-II and PSC4-II extracts on CCD 841 CoTr, HT-29, LS 180 and SW948 cells: pro-apop-totic effect of the extracts.

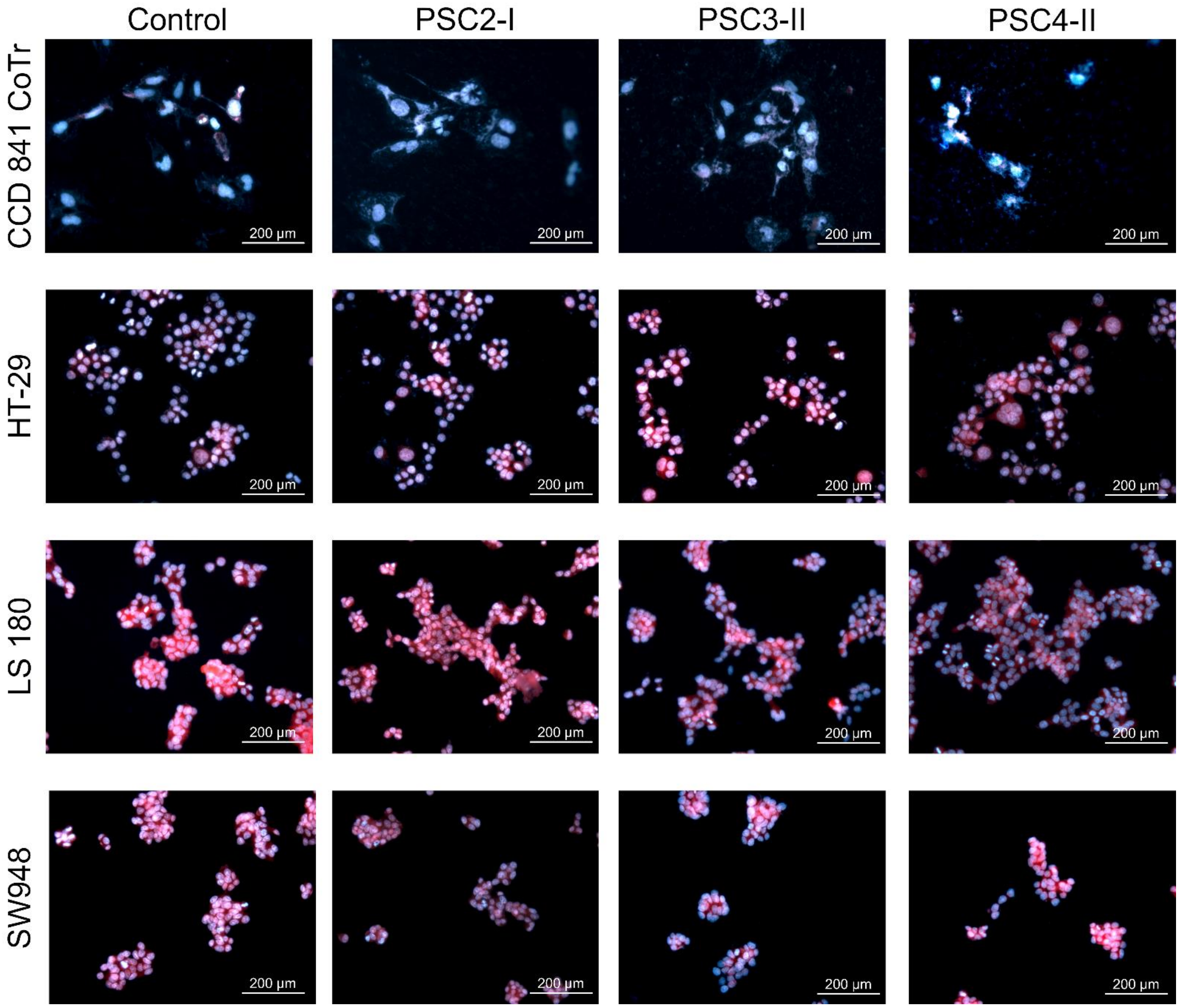

Figure S4. Anti-cancer effect of the PSC2-I, PSC3-II and PSC4-II extracts on CCD 841 CoTr, HT-29, LS 180 and SW948 cells: effect on the morphology of the cells and level of the proteins measured with DAPI/SR101 staining.

Table S2. Concentration of the NO secreted by CCD 841 CoTr, HT-29, LS 180 and SW948 cells after incubation with PSC2-I extract, with and without pre-incubation with LPS.

		PSC2-I			
		0 µg/ml	25 µg/ml	100 µg/ml	200 µg/ml
CCD 841 CoTr	LPS(-)	0.000 ± 0.000	0.000 ± 0.000	0.030 ± 0.052	0.147 ± 0.127
	LPS(+)	0.000 ± 0.000	0.000 ± 0.000	0.022 ± 0.038	0.044 ± 0.038
HT-29	LPS(-)	0.383 ± 0.037	0.748 ± 0.274	0.597 ± 0.074	0.769 ± 0.074
	LPS(+)	0.617 ± 0.126	0.642 ± 0.069	0.801 ± 0.182	0.761 ± 0.069
LS180	LPS(-)	0.451 ± 0.035	0.411 ± 0.070	0.492 ± 0.121	0.532 ± 0.070
	LPS(+)	0.564 ± 0.068	0.623 ± 0.068	0.702 ± 0.000	0.662 ± 0.068
SW948	LPS(-)	0.483 ± 0.161	0.695 ± 0.127	0.568 ± 0.127	0.949 ± 0.127
	LPS(+)	0.556 ± 0.069	0.735 ± 0.120	0.735 ± 0.120	1.015 ± 0.069

Table S3. Concentration of the NO secreted by CCD 841 CoTr, HT-29, LS 180 and SW948 cells after incubation with PSC3-II extract, with and without pre-incubation with LPS.

		PSC3-II			
		0 µg/ml	25 µg/ml	100 µg/ml	200 µg/ml
CCD 841 CoTr	LPS(-)	0.000 ± 0.000	0.000 ± 0.000	0.000 ± 0.000	0.000 ± 0.000
	LPS(+)	0.000 ± 0.000	0.000 ± 0.000	0.000 ± 0.000	0.000 ± 0.000
HT-29	LPS(-)	0.383 ± 0.037	0.297 ± 0.000	0.340 ± 0.197	0.468 ± 0.074
	LPS(+)	0.617 ± 0.126	0.562 ± 0.069	0.483 ± 0.000	0.721 ± 0.119
LS180	LPS(-)	0.451 ± 0.035	0.411 ± 0.185	0.208 ± 0.140	0.370 ± 0.121
	LPS(+)	0.564 ± 0.068	0.702 ± 0.312	0.427 ± 0.068	0.505 ± 0.136
SW948	LPS(-)	0.483 ± 0.161	0.355 ± 0.147	0.271 ± 0.073	0.440 ± 0.127
	LPS(+)	0.556 ± 0.069	0.496 ± 0.120	0.456 ± 0.069	0.536 ± 0.069

Table S4. Concentration of the NO secreted by CCD 841 CoTr, HT-29, LS 180 and SW948 cells after incubation with PSC4-II extract, with and without pre-incubation with LPS.

		PSC4-II			
		0 µg/ml	25 µg/ml	100 µg/ml	200 µg/ml
CCD 841 CoTr	LPS(-)	0.000 ± 0.000	0.000 ± 0.000	0.074 ± 0.127	0.104 ± 0.111
	LPS(+)	0.000 ± 0.000	0.000 ± 0.000	0.000 ± 0.000	0.022 ± 0.038
HT-29	LPS(-)	0.383 ± 0.037	0.554 ± 0.000	0.597 ± 0.074	0.683 ± 0.124
	LPS(+)	0.617 ± 0.126	0.681 ± 0.069	0.721 ± 0.119	1.357 ± 0.383
LS180	LPS(-)	0.451 ± 0.035	0.411 ± 0.070	0.492 ± 0.000	0.653 ± 0.070
	LPS(+)	0.564 ± 0.068	0.780 ± 0.180	0.702 ± 0.312	0.780 ± 0.068
SW948	LPS(-)	0.483 ± 0.161	0.398 ± 0.147	0.568 ± 0.127	0.630 ± 0.090
	LPS(+)	0.556 ± 0.069	0.656 ± 0.069	0.576 ± 0.183	0.895 ± 0.069
